# Community concepts of poverty: an application to premium exemptions in Ghana’s National Health Insurance Scheme

**DOI:** 10.1186/1744-8603-9-12

**Published:** 2013-03-14

**Authors:** Genevieve C Aryeetey, Caroline Jehu-Appiah, Agnes M Kotoh, Ernst Spaan, Daniel K Arhinful, Rob Baltussen, Sjaak van der Geest, Irene A Agyepong

**Affiliations:** 1 School of Public Health, University of Ghana, P. O. Box LG 13, Legon, Ghana; 2 African Development Bank, Tunis, Tunisia; 3 Radboud University Nijmegen Medical Cente, Nimegen, Netherlands; 4 Noguchi Memorial Institute for Medical Research, University of Ghana, Legon, Ghana; 5 University of Amsterdam, Amsterdam, Netherlands

**Keywords:** Health insurance, Exemptions, Poor households, Community participation, Wealth ranking, Targeting, Ghana

## Abstract

**Background:**

Poverty is multi dimensional. Beyond the quantitative and tangible issues related to inadequate income it also has equally important social, more intangible and difficult if not impossible to quantify dimensions. In 2009, we explored these social and relativist dimension of poverty in five communities in the South of Ghana with differing socio economic characteristics to inform the development and implementation of policies and programs to identify and target the poor for premium exemptions under Ghana’s National Health Insurance Scheme.

**Methods:**

We employed participatory wealth ranking (PWR) a qualitative tool for the exploration of community concepts, identification and ranking of households into socioeconomic groups. Key informants within the community ranked households into wealth categories after discussing in detail concepts and indicators of poverty.

**Results:**

Community defined indicators of poverty covered themes related to type of employment, educational attainment of children, food availability, physical appearance, housing conditions, asset ownership, health seeking behavior, social exclusion and marginalization. The poverty indicators discussed shared commonalities but contrasted in the patterns of ranking per community.

**Conclusion:**

The in-depth nature of the PWR process precludes it from being used for identification of the poor on a large national scale in a program such as the NHIS. However, PWR can provide valuable qualitative input to enrich discussions, development and implementation of policies, programs and tools for large scale interventions and targeting of the poor for social welfare programs such as premium exemption for health care.

## Background

Poverty is a concept that is not easily defined and measured. Identifying poor for any social intervention are usually challenging with regard to accurately targeting eligible beneficiaries on the basis of poverty. The quantitative means testing approach, related to a more positivist knowledge paradigm has been used to determine poverty levels based on identified income or consumption expenditures. This approach premises that to be poor is to be lacking in some material worth, to be deficient in some way, or to fail to meet a ‘minimum requirement’ of something [1,2]. In contrast the qualitative approach premises that poverty is a social construct whose understanding requires a contextual and relativist approach to knowledge [3-5]. From this perspective and to borrow the words of Gilson et al. (2011), poverty is to some extent ‘constructed and brought alive by social actors through the meaning they attach to the interpretation of their experience’[6]. In response to broadening the dimensions of poverty the 2000/2001 World Development Report encompassed multiple dimensions of poverty, going beyond material deprivation to other forms of deprivation [7]. It broadened the notion of poverty to include vulnerability, insecurity, voicelessness and powerlessness. The report defined poverty as ‘the lack of or the inability to achieve a socially acceptable standard of living’. Subsequently mixed methods have emerged exploring both qualitative-quantitative dimensions of poverty to provide a comprehensive view on poverty [8-10].

Since 2004 Ghana has been implementing a National Health Insurance Scheme (NHIS) to replace out of pocket fees at point of service as a more equitable and pro-poor health financing policy. The NHIS is publicly financed by a national health insurance fund. The fund has three main sources. The first, making up about 70% of the fund is a 2.5% value added tax (VAT) known as the National Health Insurance Levy. The second, making up about 20 – 25% of the fund is 2.5% of contributions of Social Security and National Insurance Trust (SSNIT) contributors who are predominantly public and private formal sector employees. Because of the direct income deductions SSNIT contributors do not pay an out of pocket premium. The third is out-of-pocket premiums ranging between GH¢7 ($5) to GH¢48 ($34) from the non SSNIT contributors – who are mainly informal sector workers. In theory non SSNIT contributor out of pocket premiums are income adjusted. In practice to assess non formal sector incomes is almost impossible and many district scheme offices simply apply a flat rate. Additionally everybody, whether SSNIT or non SSNIT contributor pays an annual registration fee of approximately GHC 4 ($2). The minimum premium for exemptions is GH14 ($10). Ensuring equity in enrollment though identification of and premium exemptions for individuals and groups without adequate financial resources to pay referred to in the NHIS law (LI 1809) as indigents or the “poorest of the poor”, is one of the stated goals of the NHIS.

In the rest of this document, we will use the terminology of Ghana’s National Health Insurance law of “indigents” and the terminology of “poorest of the poor” or simply “the poorest” interchangeably. We will use it in the meaning intended to our understanding by the Ghanaian policy of individuals and groups whose financial resources are inadequate or so limited that making out of pocket payments for essential services (in this case health insurance) will compromise basic survival needs such as food, clothing and shelter. We will also distinguish between the term “poverty” which refers to the condition visibly expressed by inadequate material and financial resources in relation to the needs of daily living and survival in a particular context; as distinct from the terms “indigents”, “poorest of the poor” and “poorest” used interchangeably to refer to people living in the extremes of this condition of poverty.

The effective realization of the goal of exempting the poorest of the poor from out of pocket health insurance premium payments remains a major challenge in part because of difficulties in identifying and therefore being able to targeting this group. This problem of identification is not new, and has beset the implementation of other policies targeting groups that may have difficulty in paying out of pocket fees for essential services in Ghana [11-14].

According to the Legislative Instrument (LI 1809) that accompanied the NHIS, a person shall be identified as an indigent and exempted from premium payments under four main criteria. These are (i) that the person is unemployed and has no visible source of income, (ii) does not have a fixed place of residence according to standards determined by the scheme, (iii) does not live with a person who is employed and who has a fixed place of residence and (iv) does not have any identifiable consistent support from another person [15,16]. Observation and experience over the years since LI 1809 was passed suggests that effectively these criteria do not identify the poorest of the poor in Ghana. Hardly anyone qualifies for an exemption with their strict application despite observations that many are not enrolled because of difficulties in paying the non SSNIT contributor out of pocket registration fees and premium.

It is in this light that the study set out to identify and compare various strategies to identify the indigent for premium exemptions. In a previous paper, we have analyzed the issues around identifying the poor for premium exemptions from a more quantitative perspective by comparing estimates of the effectiveness and efficiency of means testing (MT), proxy means testing (PMT), participatory wealth ranking (PWR) and geographic targeting (GT), in communities with different socio economic characteristics[17]. Whereas MT is considered an absolute quantitative measure of poverty (by using a poverty line), PMT and PWR are relative measures of poverty. PMT classifies households in five equal wealth quintiles based on selected quantifiable assets and PWR based on community perceptions on poverty. This prevents a direct comparison of all strategies, as PMT and PWR do not provide a clear cut-off level on who can be classified as the poorest of the poor or “indigent”. Therefore, we assumed in the baseline analysis for PMT that the bottom 40% of all households (i.e. the lowest two quintiles) represents “indigent” households, and for PWR that all households labeled as ‘very poor’ and ‘poor’ represent “indigent” households. The analysis showed that mean testing and proxy means testing correlated more closely with each other in the households they identified as the poorest of the poor than they did with the more relativist participatory wealth ranking. In this paper we explore in more depth the qualitative and relativist indicators of poverty revealed by the participatory wealth ranking process. The depth and ‘thickness’ of insights provided in this process are lost in the more quantitative approaches.

Participatory Wealth Ranking has been widely used to promote discussions on locally relevant dimensions of poverty and other community interventions [3,18-22]. It is based on the concept of utilizing local knowledge about the levels of relative poverty and wealth. Key informants rank their fellow community members into wealth categories after they have discussed in detail underlying concepts of poverty and wealth within their communities. The process involves three main stages of mapping, ranking and analysis [23]. In this study we explored community indicators of poverty, and the implications and potential usefulness of using PWR to identify the poor for premium exemptions as compared to quantitative measures in the context of Ghana, a lower middle income country in sub-Saharan Africa.

## Methods

### Study setting

We conducted the study in five communities of the Central Region of Ghana in June 2009. The region was purposively selected for the study as one of the regions of Southern Ghana whose poverty, infant and child mortality statistics are relatively poor. It has 17 administrative districts classified as rural, urban and semi-urban. The urbanized population constitutes around 37.5% and is clustered in the three districts of Cape Coast, Awutu and Agona. The region is predominantly Akan speaking (82.0%), majority being ‘Fantes’ who are indigenes. The rest include Guan (6.1%), Ewe (4.8%) and a number of small ethnic groups of migrants comprising Mole Dagbon, Grusi; Gurma and Mande- Busanga, constituting 3.4% of the population, from the northern part of the country. Agriculture and fishing are the main occupations within the Region and employs more than two thirds of the work force in many districts. Cocoa production is concentrated in northern parts of the region while oil palm production predominates in north and central parts of the region. Cape Coast, the regional capital, has the highest proportion of formal sector workers with a mix of artisans and traders [24].

In 1999 the region had a poverty incidence rate of 48% which was above the national average of 39%. However, it experienced rapid decline in poverty incidence between 1999 and 2005 from 48% to 20%. This was consistent with the overall decline in poverty across the country from 39% to 28.5%.[ref: GSS 2007] Nevertheless, for many the lack of money to pay for health services, distance to health facilities and transport cost were some factors identified as limiting access to health care in the region. With regard to NHIS enrollment 23% of people within the region were enrolled (holders of valid insurance ID cards) at the time of the study. This rate represents the lowest participation in the scheme in comparison to other regions of the country [25]. Enrollment rate by socioeconomic quintiles revealed 21% of households within the poorest quintile (Q1) were enrolled compared to 37% within the richest quintile (Q5) [26].

### Selection of study communities

In selecting our study communities, we assumed that community perceptions of poverty might differ by poverty incidence, socio economic, socio cultural and rural urban characteristics. We therefore used a purposive multistage sampling of first selection of districts based on poverty incidence (PI) followed by selection of communities within the districts. We used data from the estimated district poverty incidence (PI) for Ghana in 2005 [27] to select the district with the lowest poverty incidence (Upper Dankyira, PI 26%) and the district with the highest poverty incidence (Gomoa, PI 63%). We selected Cape Coast metropolis (PI 27%) because it was the region’s only metropolitan district. Based on predominant economic activities, we selected a fishing district (Mfantsiman, PI 47%) and a farming district (Assin North, PI 29%).

To select communities from the districts, we obtained a list of the most recent (i.e. 2000) census enumeration areas (EAs) with their populations, from the Ghana Statistical Service. Enumeration areas comprise sections of communities containing 150–200 dwelling structures that have been mapped out by the Ghana Statistical Services for census purposes. The EAs are classified by the Ghana Statistical Services into rural, urban and semi-urban depending on the population density and other demographic features [28]. For the purposes of our study, an EA was considered as a community. In the rest of this article EA and community will be used interchangeably. From the low poverty incidence district, we selected a rural EA; from the high poverty incidence district, a semi urban EA; from the farming district, a semi urban EA and from the fishing district, an urban EA. Finally, we selected one urban EA from the Metropolitan district. Therefore, five study communities were selected for this study.

### Community entry and selection of key informants

We made initial visits to the communities to inform the chief (traditional leader) and community leaders about the study and request permission to work with some community members who would serve as our key informants. We requested that key informants should include opinion leaders, leaders of associations within the community, persons who have lived long enough within the community to know everyone and anyone who could articulate the views of the community. We also requested that the key informants should be a mixed group of men and women from varied socioeconomic backgrounds. In all the communities, the chief appointed a key community representative, usually the *Assembly Man* (elected local government representative) to assist in the selection of key informants. Detailed demographic characteristics of key informants are provided in Table 1. The entire community was also informed through community radio networks and in some cases, the beating of the *gong*-*gong* (a local metal bell traditionally used to assemble people to give information).

**Table 1:** Characteristics of key informants

**Indicators/District**	**Metropolitan**	**Rural**	**Semi-urban**	**Typically farming**	**Typically fishing**
Poverty incidence	27%	26%	63%	29%	47%
Major economic activity	Trading	Farming (cash crop)	Farming(food crop)	Farming (cash crop)	Fishing
Number of households	186	179	161	109	105
Number of key informants	22	18	16	18	18
***Characteristics of key informants (%)***					
Males	17 (77.2)	11 (61.1)	9 (56.3)	7 (38.9)	8 (44.4)
Females	5 (22.7)	7 (38.9)	7 (43.7)	11 (61.1)	10(55.6)
Minimum age	27	32	28	20	25
Maximum age	70	72	62	65	70
Average age	49	51	46	43	42
***Education***					
None	4 (18.2)	2 (11.1)	3 (18.7)	3 (16.6)	8 (44.4)
Primary	2 (9.1)	1 (5.6)		5 (27.8)	3(16.7)
Middle/JHS	10 (45.5)	12 (66.6)	13 (81.3)	8 (44.4)	7 (38.9)
Secondary	3 (13.6)	3 (16.6)		1 (5.56)	
Tertiary	3 (13.6)			1 (5.56)	
***Main occupation***					
Fisherman					7 (38.9)
Fishmonger					4 (22.2)
Farmer	4 (18.2)	15 (83.3)	7 (43.8)	8 (44.4)	1 (5.6)
Trader	4 (18.1)	1 (5.56)	7 (43.8)	8 (44.4)	1 (5.6)
Artisan	6 (27.3)	2 (11.1)	1 (12.5)	1 (5.6)	3 (16.7)
Laborer	2 (9.1)				2 (11.1)
Professional	4 (18.2)			1 (5.6)	
unemployed	2 (9.1)		1 (6.25)		
***Marital status***					
Single	1(4.55)			1 (5.6)	2 (11.1)
Married	17 (77.27)	16(88.89)	8(50)	12 (66.6)	9 (50.0)
Divorced/separated	3 (13.64)		6 (37.5)	2 (11.1)	3 (16.7)
Widow(er)	1 (4.55)	2(11.11)	2 (12.5)	3 (16.6)	4 (22.2)
***Religion***					
Christian	7 (31.8)	18 (100.0)	14 (87.5)	15(83.33)	17 (94.4)
Muslim	14 (63.6)		2 (12.5)	3 (16.67)	
None	1 (4.55)				1 (5.6)
Belongs to an association	5(22.73)	5(27.78)	4(25)	4(22.22)	11(61%)

### Research personnel and community consent

All authors participated in the study design and analysis. The participatory wealth ranking process was conducted by the lead author with two facilitators with experience in organizing focus group discussions. The facilitators went through one week of training to familiarize with the concept of participatory wealth ranking, coordination of discussions and recording in line with our study objectives. Our research project received ethical clearance from the Ghana Health Services Ethical Review Committee. We obtained written informed consent from literate respondents and verbal consent from the non-literates. All discussions were held in the local languages and were recorded on flip charts, audio recorded and later transcribed.

### Steps in participatory wealth ranking

a. *Mapping.* A community map was constructed and all households within the boundaries of the community listed. To facilitate this process, we obtained enumeration area (EA) maps from the Ghana Statistical Service to delineate the community boundaries. All addresses of dwelling structures within the delineated boundary of the community and names of household heads were obtained to generate a household list.

b. *Ranking.* This process involved key informants identifying dimensions and indicators of poverty from which households were ranked. At our first meeting with the key informants, we explained the purpose and objectives of our study. We then led discussions to investigate community dimensions of poverty and wealth by posing the question to the informants: What distinguishes a rich household from a poor household? We guided key informants to develop a chart listing all indicators for the two categories in the community and subsequently further classify the two categories into a continuum of five categories of very poor, poor, middle class, rich and very rich. Within each category, the informants identified and discussed in detail the characteristics that closely described households. The informants received an envelope containing colored cards, representing the five categories. The informants were divided into three groups with a facilitator assigned to the group and a copy of the household list. After informants were sufficiently instructed on procedure, the name of the household head was read out, and informants raised the color card they felt best classified the household. The colors were raised simultaneously, counted and recorded by the facilitator for each group. No discussion was allowed among informants during the ranking process to ensure that their decisions were as independent as possible. Anyone who indicated they did not know a particular household was not allowed to participate in the ranking of that household.

c. *Analysis.* To estimate household wealth status, we first tallied the number of each color card assigned by the informants to the household. The decision on the household’s wealth status was based on a simple majority of the number of same color cards assigned by the informants. If a household received equal numbers for two different color categories the overall distribution of card assignment was examined. If the distribution curve was skewed in a particular direction, the household was given a wealth status in the direction of the skew. We calculated a simple household socioeconomic score by assigning values to the cards as follows: very rich (yellow, five (100%)), rich (blue, four (80%)), middle class (green, three (60%)), poor (pink, two (40%)) and very poor (orange, one (20%)). We present part of a table for one of the communities to illustrate (Table 2).

**Table 2:** An example participatory wealth ranking result

**Household**	**Very Poor (1)**	**Poor (2)**	**Middle class (3)**	**Rich (4)**	**Very Rich (5)**	**Score**	**Decision**
Household 1	0	8	10	0	0	60	Middle Class
Household 2	0	7	7	4	0	60	Middle Class
Household 3	1	13	5	0	0	40	Poor
Household 4	5	6	6	1	0	40	Poor
Household 5	10	7	0	0	0	20	Very Poor
Household 6	0	0	2	15	1	80	Rich
Household 7	0	0	2	6	10	100	Very Rich

## Results

Several indicators consistently came up in the key informant discussions in the five communities to assess the socioeconomic status of households. In all but the metropolitan community, poverty was defined as *ohia* while extreme poverty was defined as *ohia buroburoo* or *ohia nemi-nemi*. In the metropolitan community, the term *ahokyir* was used to define poverty while *ohia* without any qualification was reserved to describe extreme poverty. *Ahokyir* translates as a state of being in a tight and difficult position and can be used in reference to any difficult situation rather than exclusively to poverty. *Ahokyir* as applied to poverty in the urban context was defined as being financially tight, having a difficulty in making ends meet, as a temporary acute state. A person in *ahokyir* can sometimes borrow money and repay at a later date. Extreme poverty on the other hand, defined simply as *ohia* in the urban context, represented those who consistently go through financial difficulty on long term chronic basis. “Ohia” in the urban context appeared to mean a similar thing to “ohia buburoo” or “ohia nemi nemi” in the rural and semi-urban study contexts.

Since our study focus is on poverty and identification of the “poorest” we present below the discussion on the socioeconomic indicators of poverty and the poorest and represent the corresponding indicators for the non poor in a table in the appendix. In the conceptualization the community, refers to poverty as a condition visibly expressed by material resource and financial difficulty. It is a condition, which has variation or degrees from mild to extreme i.e. “ohia” versus “ohia buroburoo” or “ohia nemi nemi”; but could also be a temporary condition as in the “ahokyir” of the urban context; or a more chronic enduring one. The poorest are the individuals and groups at the extreme end of this condition of poverty.

### Employment

Apart from the fishing and metropolitan communities, the dominant economic activity for the other three communities was farming. The poorest were involved in subsistence farming usually on other people’s land as wage laborers. For those who owned land, the land sizes were normally less than one acre. In one farming community, informants also categorized palm nut crackers as among the poorest because they did not have farm lands, were neither employed as farm laborers and the returns for cracking palm nut for oil are low. The informants in another farming community linked poverty in their community to laziness especially of persons who are strong and healthy. The poorest were allegedly not willing to farm to take care of themselves and their families despite that one could work on other people’s land on a convenient agreement (Table 3).

**Table 3:** Employment and assets ownership indicators

	**Categories/Communities**	**Metropolitan**	**Rural**	**Semi urban**	**Typically farming**	**Typically fishing**
	**Employment**					
**Employment**	a. Very poor	a. Unemployed; by day farm laborer	a. Beggars	a. Beggars	a. By-day farm laborer	a. Fish and water porters
	b. Poor	b. By day laborers; child labor to supplement family income	b. A laborer; thief; lazy person	b. A farm laborer	b. Mobile tailors, by day construction workers	b.Kitchen assistants
	c. Middle class	c. Artisans; informal self-employee; formal sector employees	c. Farmer able to harvest up to 5 bags of cocoa annually; mobile goods retailer	c. A farmer able to hire laborers; an artisan; a taxi driver	c. Retailers, artisans, drivers, traders, teachers	c. Mini retail services, artisans, salaried workers
	d. Rich	d. Retail and wholesale business	d. Farmer able to harvest up to 10 bags of cocoa annually	d. A farmer able to hire laborers; owner of a drug/agro-chemical store	d. Wholesalers, drug store/agro chemical shop owners	d. Contractor involved in wholesale business
	e. Very rich	e. Commercial trader with agglomeration of stores; medical doctor; building and road contractors; money lender	e. A farmer-able to harvest cocoa up to 40 bags a year; salaried workers	e. Owns and operate a private school	e. Commercial vehicle owner, money lender	e. Owns a boat with employees
	**Assets Ownership**					
**Assets Ownership**	a. Very poor	a. No assets	a. No assets	a. No assets	a. No assets	a. No assets
	b. Poor	b. Radio	b. Less an acre of land	b. Less than an acre of land for farming	b.1 piece of cloth; 1 ½ acre of land; radio, goats	b. Radio
	c. Middle class	c. Radio, DVD players, living room furniture, TV	c. Has a mobile Phone	c. 2 acres of land for farming, fridge, stores, TV	c. 3 acres of land for cocoa farming, brick house, TV, radio, fridge	c.½ plot of land; fridge; has a 3 bedroom house; set of furniture
	d. Rich	d. Has 1 car; Has about 6 taxis working for him	d. A retail shop; 5–6 acres of cocoa farms	d. Radio, mobile phone, TV, audio system for hiring, land (10 acres)	d. Corn mill; commercial vehicles; 4 acres cocoa farm, 4 acres of palm plantation, 3 acres of orange farm	d. Personal boat for fishing
	e. Very rich	e. Saw mill/flour mill	e. Above 10 acres of cocoa farms	e. Compound houses for hiring, a private school; mobile phone; over 10 acres of land	e. 12 acres of cocoa land, 6 acres for palm plantation; drug store, chain saw for commercial purposes	e. Employed other fishermen working with other boats owned

In the metropolitan area, informants indicated that the poorest were usually unemployed or engaged in work that yielded minimal wages such as construction site laborers. In addition the poorest households sent their children to sell commodities on the streets for supplementary household income. In the fishing community where simple traditional methods of fishing with canoes continue to be widely used, informants described poverty as a seasonal phenomenon exacerbated by the unpredictability of the weather.

‘Poverty is a cycle in this community. We become better off during the bumper fishing season and retreat into poverty when the season is over. Even though we are currently in the fishing season, yields are low and we sometimes come back with an empty boat. The weather patterns have changed and we cannot predict with accuracy any more’. (Key informant fishing community)

### Education of children

The ability to afford children’s education to the highest level, the school a child attended and educational materials available to a child were mentioned as indicators of household wealth. Community key informants observed that in general the poorest households were unable to afford education of their children beyond the primary level (primary 6). The children of the poorest families usually attended public schools in contrast to the more wealthy who sent their children to private schools. Children of the poorest households also had fewer books to study with, and often only had a single notebook for all subjects. In the metropolitan community, children from the poorest households attended a community established school known as *onkaakyir* (meaning it is never too late) for which parents do not pay school fees but pay GH¢1(US$0.70) as administrative charges. In the fishing community key informants indicated that the poorest parents did not allow their sons to obtain any formal education but rather let them accompany and assist them in their fishing activities. They explained

‘Fishing brings in quick money compared to education. When a child goes to school, it takes a long time before he is able to make money. Sometimes when they are unable to find jobs, they resort to fishing anyway. It is also better to train them when they are young so they can take over from their parents’. (Key informant fishing community)

### Food availability

The number, quality and variation of meals were characteristics mentioned that described household’s wealth status. The poorest were unable to afford three meals daily and meals were often made up of cooked cassava with a little palm oil and salt without vegetables or fish. A typical meal of the poor was roasted corn called *nkyewie*ε with water. On a favorable day, they ate *gari* (cassava grated, dried and fried in base of palm or vegetable oil) for dinner, which is affordable and has the capacity to sustain a person for long hours after eating. The poorest usually ate one heavy meal made up of gari in the afternoon to sustain them for the rest of the day. Some begged for ingredients such as oil, tomatoes and pepper from neighbors to cook the family dinner.

### Physical appearance

Informants indicated that it was common to see the poorest wearing miss-matched old plastic bathroom slippers and worn out, often dirty clothes. Their children wore few clothes and tended to go around half naked. An informant from the semi-urban community commented that at Christmas, the poorest households bought used clothes (*obroni wawu*) for their children to attend church. Others compared the appearance of the poorest persons to that of a mad person: they always look unkempt, wear mismatched old slippers and very often beg for food. The poorest could not afford change of clothes and did what was termed in the local parlance as ‘*wash and wear’* that is after washing their single clothes they wait until dry and put them back on. They also borrowed clothes from others to attend social functions such as funerals.

### Assets ownership and housing

The poorest were characterized by limited or total absence of even the simplest of assets within their households. They usually only had in their possession a small radio, a mat and some cooking utensils. Their dwelling structures were made of mud with thatched roofing, usually a single dwelling. Bathroom and toilet facilities were shared with other households.

### Health seeking behavior

The key informants indicated that the poorest often found it difficult to pay for modern health care related expenses such as medicines, consultation, admissions, and transport to health facilities. Because of this, they depended mostly on herbal treatments at home or sought healing from traditional healers including fetish priests (*Okomfo*). Those who could afford it purchased pain killers and blood tonics from the local drug stores to treat common ailments without consulting a doctor.

### Social exclusion and marginalization

Social exclusion consists of dynamic, multidimensional processes driven by unequal power relationships interacting across four main dimensions (economic, political, social and cultural) and at different levels including individual, household, group, community, country and global levels. It results in a continuum of inclusion/exclusion characterised by unequal access to resources, capabilities and rights which leads to health inequalities [29] The poorest people according to informants were usually not included in group decision making processes either at the family or community level. Informants explained that families meet when they were bereaved or on other social occasions such as festivals. During such meetings, members contributed money. As the poorest were unable to contribute money, their suggestions were not considered and their concerns usually not addressed. In the words of a key informant: ‘*Ohianii ye obi a yenfr*ε *no nka nipa ho’* (A poorest person is one whose opinion does not count in group decision making because he is not called or ignored when present) Table 4.

**Table 4:** Health and social exclusion indicators

	**Categories/Communities**	**Metropolitan**	**Rural**	**Semi urban**	**Typically farming**	**Typically fishing**
	**Health Seeking Behavior**					
**Health Seeking Behavior**	a. Very poor	a. Cannot afford transport cost to health center	a. Use herbs to treat sickness	a. Cannot meet medical expenses-use herbs	a. Uses leaves and plants from the forest to cure sickness	a. Cures himself with herbs from nearby bushes
	b. Poor	b. Self-medication	b. Use herbs to treat sickness	b. Cannot meet medical expenses and transport cost to hospital	b. Buys pain killers from drug stores; seek help from traditional healers	b. Buys medicine from chemical sellers
	c. Middle class	c. Able to meet out- patient medical expenses	c. Able to meet out-patient medical expenses	c. Able to meet out-patient medical expenses	c. Attends hospitals and private clinics	c. Able to attend hospital or nearby health centers
	d. Rich	d. Able to afford health care cost	d. Able to afford health care cost	d. Able to afford medical cost for entire family	d. Goes for regular medical checkups, attends private hospitals	d. Sometimes have personal doctors to attend to their health needs
	e. Very rich	e. Visits the hospital when sick and able to pay medical bills	e. Visit the hospital and able to meet medical bills	e. Able to afford health care cost for family	e. Have special doctors to attend to their needs	e. Usually goes for regular medical check-ups
	**Social Exclusion and Marginalization**					
**Social exclusion and Marginalization**	a. Very poor	a. People pay less attention to their needs and opinions	a. Humiliated in the open especially if they are unable to repay their debtors	a. They are laughed at and teased by children; their views are taken for granted	a. Excluded from family/social gatherings and meetings	a. They are considered outcast and not given any recognition within the community
	b. Poor	b. They are publicly insulted and laughed at by adults and children	b. Children are given severe punishment than their non-poor counterparts at school	b. Teachers do not give children from poor households enough attention compared to non poor children	b. Invited to social gatherings but views are not considered important	b. Usually laughed at because of their appearance
	c. Middle class	c. Their opinions are generally accepted and respected	c. They are not disregarded nor laughed at in public	c. Their views are usually accepted as the general rule within the community	c. Always part of meetings and sometimes serve as the voice gap between the rich and poor	c. Their views are respected as they are able to speak in favor of the poor sometimes
	d. Rich	d. Their views are always accepted and they command a lot of respect	d. They are respected and sometimes treated as chiefs	d. They receive many accolades especially during the Muslim festivals	d. Usually consulted before decisions are taken in the family/community	d. They coordinate social gatherings and have authority to veto decisions
	e. Very rich	e. They are highly respected, their opinions on decisions are regarded as final	e. Wherever they go, they are accorded the necessary attention and audience	e. They receive similar treatment and respect as their rich counterparts	e. They command respect and given high positions at church and other social gatherings	e. Highly respected members of the community

## Discussion

Our participatory wealth ranking (PWR) process has provided a qualitative understanding of community defined indicators of poverty in Ghana. The process and its findings are of importance for several reasons. First it highlights the fact that given the social and contextual nature of poverty rigid application of over simplified and standardized lists of identification criteria are not the way to go. Poverty is best identified by a composite of variables rather than one or a few simple (monetary) variables [8,30,31]. Our study revealed that with regard to material indicators of poverty, the range of indicators covered the themes of employment, ability to put and keep children in school, food availability and consumption, physical appearance, housing conditions, asset ownership, and health seeking behavior. With regard to the more non material indicators, social exclusion and marginalization was a recurring theme. From our key informant perspectives, a combination of these indicators rather than any single one defined the poorest households. Indeed in comparing similar indicators from our household survey of the same communities, we observed that households within the lower quintiles had lower levels of education, high levels of unemployment, limited number of assets and poor housing conditions.

Second, the community defined indicators of poverty were different from the indicators applied as criteria for exemptions under the National health insurance law in Legislative Instrument (LI) 1809. The defined criteria of unemployment with no visible source of income; no fixed place of residence according to standards determined by the scheme; not living with a person who is employed and who has a fixed place of residence and not having any identifiable consistent support from another person are clearly inappropriate from a community perspective in all the rural and semi urban sites. It was only in the urban community that unemployment was mentioned an indicator of poverty. In all the other communities, the poorest were employed predominantly in the traditional methods of farming and fishing which yielded lower remuneration.

The process of the national health insurance legislation development was a highly centralized one, often dominated by a small group of policy elite [32]. These sharply divergent criteria and perspectives on poverty between the national level policy elite who developed the Legislative Instrument and community members bring to the front the inappropriateness of purely centralized approaches to designing social reform policies and legislations for the poorest. In developing such policy and accompanying programs, it is important to engage communities and make sure an understanding of their situation and perspectives informs the process. This will require special strategies targeted at getting the voice of the poorest, given the observations from our study of the social exclusion and marginalization of the poorest in community decision making.

Third, the ranking patterns of who is poor varied widely across communities as perceived state of poverty was relative to the context of the communities. In the typically farming (cash crop farming) community for example, households were perceived and ranked predominantly with middle class status contrasting extremely poor perceptions and ranks assigned to households in the typically fishing community. These perceptions did not overlap very well with the purely material indicators of poverty from our accompanying quantitative work that compared number of poor households from PWR versus MT. Clearly the more quantitative measures are measuring somewhat different dimensions of poverty as compared to PWR [17,19]. The question that arises is which of these measures is of more importance in addressing poverty? Are households who are perceived in the context of the communities as “poorest” indeed the poorest regardless of where they fall on a means test? In a social context, perceptions and appearances can have more influence on the experience of individuals and households than their poverty or lack of it as measured on a quantitative scale such as means testing. There is a need to further explore how perceptions of the wealth and poverty status of households by their community members affect their experiences and vulnerability regardless of their actual material means.

Fourth, marginalization and social exclusion are also critical in the understanding of poverty in the study context. It is of interest that several of our key informants raised the issue of the poorest not being taken seriously or being ignored when community or family meetings are organized. The poorest are therefore the silent ones in society whose suggestions and contributions are considered, if ever, with less importance. The finding confirms earlier one by Van der Geest (1997) [33] in a study of money and respect among the elderly in a rural community in Ghana that having money measures the quality of the elderly with regard to the respect and prestige they receive within the community. Being poor was described as being ‘useless’ and lacking respect. Such notions about the poor is conveyed in the Akan proverb that *Ohiani bu bε a yεnnfa*; literally translated “a poor man’s proverb does not spread” [34]. The deep meaning is that no one takes notice of what the poor says. That perception of the poor certainly points out a significant dilemma of poverty; those who are poor and more likely to require social support for health care are also the more likely to be ignored because they are the voiceless.

A number of key issues are important in interpretation of our results: first, in our current study, the indicators identified showed similarities across communities. The study was conducted in one region and there could be some variation when applying PWR to another region like the north with higher poverty levels and differences in socio cultural context and lifestyles. Second, the use of subjective measures implies that the definition of who is poor may vary widely across settings, as sometimes people’s expectations about benefits of the identification process and variations in relative perceptions of “poverty” may exaggerate or underestimate the numbers of identified poor. This may provoke in some way unequal exemption policies in a country like Ghana. Hence, the acceptability of PWR, in the population and among policy makers, as a uniform strategy to identify the poor in Ghana may be questionable. Third, the implementation of PWR is more feasible in terms of community interest, time and ease of administration in rural communities than urban. Rural communities are generally more are closely knit, and people know each other well to ascertain their poverty status. In this regard, our observations are in conformity with similar studies where PWR and other programs for the poorest have been carried out mainly in rural communities [19,22]. Fourth, community members who represent the key informants are crucial in the outcome of the results. If there are imbalances in the representation, it may influence the results in ways that will not ensure true ranking of households.

## Conclusion

Participatory wealth ranking has the advantage of providing qualitative details and relative perspectives, which are otherwise missing in quantitative measures such as means testing and proxy means testing. Using community based concepts of poverty could strengthen quantitative measurement, in terms of aptness and social acceptability. The findings here show that PWR exercise help to remind us that the communities are primary stakeholders in defining who their poorest are and so decisions to help them stand to gain if they are part of the processes as in the case here the decision to implement premium exemptions. In a large scale program such as national health insurance it may not be possible to apply PWR in every community. However, if the outcome of the participatory wealth ranking process in several selected communities considered as representative of an entire district for example, yields similar indicators, these indicators can become of assistance in constructing a more appropriate tool for large scale identification of the poor. Our study holds relevance for other countries and programs struggling with the challenging issues related to identification and targeting of the poor for social welfare programs.

## Competing interest

We declare no competing interest in preparation and submission of this article.

## Authors’ contribution

GCA main author responsible for design, analysis, interpretation and write up of the manuscript; CJA involved in drafting and revision of manuscript; AMK provided further anthropological perspectives on poverty and reasons for low enrolment among the poor; ES involved in critical revision of manuscript; DKA involved in qualitative data collection, design, and critical revision of manuscript; RB involved in conception and critical revision of manuscript; SVDG involved in conception, design and revision of manuscript; IAA involved in concept, design, interpretation of data and revision of manuscript. All authors read and approved the final manuscript.
